# Fish response to the presence of hydrokinetic turbines as a sustainable energy solution

**DOI:** 10.1038/s41598-023-33000-w

**Published:** 2023-05-08

**Authors:** Stephanie Müller, Valentine Muhawenimana, Guglielmo Sonnino-Sorisio, Catherine A. M. E. Wilson, Joanne Cable, Pablo Ouro

**Affiliations:** 1 School of Engineering, Cardiff University, The Parade, Cardiff, Wales CF24 3AA UK; 2 School of Biosciences, Cardiff University, Park Place, Cardiff, Wales CF10 3AX UK; 3 School of Engineering, University of Manchester, Oxford Road, Manchester, England M13 9PL UK

**Keywords:** Energy infrastructure, Renewable energy, Animal migration

## Abstract

Hydrokinetic turbines such as vertical axis turbines (VATs) may provide decentralised, clean, sustainable energy for remote communities that lack access to the main energy grid or renewable resources. As traditional hydropower adversely alters aquatic ecosystems, it is essential to evaluate the environmental consequences of deploying VATs in riverine ecosystems to meet current and future energy needs. This study explores the implications of VATs on fish movement by observing fish swimming behaviour under two discharges, turbine operation states, and cross-sections confinements using scaled laboratory experiments. Our findings reveal that for cross-sectional confined conditions neither discharge, turbine presence, nor device operation, prevented fish from passing around and through the turbine both in the up- and downstream directions. However, fish spent the least time near the turbine vicinity and within the turbine’s turbulent, low-velocity wake, indicating avoidance behaviour. Swimming in a less confined test section further reduced the time spent within the turbine’s vicinity and wake, increasing the distance fish kept away from the device. Our results contribute to an understanding of VATs as low-risk hazards for fish swimming behaviour, advancing the potential of deploying VATs in rivers, estuaries or sea as a renewable energy solution for remote communities.

## Introduction

Currently, almost 800 million people lack access to affordable, reliable, and sustainable energy supply, resulting in social-economic inequality^1^. Particularly affected are low-to-middle-income countries, small islands, and landlocked countries, with sparsely distributed populations, often consisting of numerous remote communities, most without access to clean energy. The United Nations (UN) Agenda for Sustainable Development aims to reduce this injustice by ensuring efforts are made to provide universal access to clean energy by 2030 (Sustainable Development Goal 7)^1^, thus requiring an increase in renewable energy share.

Countries rich in large water resources and therefore ideal locations for hydropower applications, are often hotspots for biodiversity that needs protecting and conserving. The Amazon, Congo, and Mekong basins, for instance, are home to one-third of the world’s freshwater fish species, most of them unique to their geographical location^2^. Conventional hydropower schemes often utilise dams and weirs, diverting or impounding river flow to create reservoirs and head differences, thus contributing to the fragmentation of rivers. The numerous hydraulic structures installed along the rivers and rapid changes to the aquatic environment not only create migration barriers to aquatic organisms but also limit the transport of energy and sediment^3–6^, which caused, for instance, the near extinction of 4–10% of fish species in South America^7^.

To minimise dependency on conventional hydropower, the potential of all types of hydropower technologies must be explored using innovative solutions. A study of the the Brazilian Amazon, for instance, showed that in-stream turbines have the potential to cover approximately 63% of the total energy planned to be installed in form of conventianl hydropower^8^. Hydrokinetic, in-stream turbines, such as vertical axis turbines (VAT), harness the kinetic energy of the free-flowing river flow without diverting it or requiring a hydraulic structure to generate a head difference. These turbines may be transported as an assembled unit or as parts and be assembled on site and can be easily installed in any river section. Hydrokinetic turbines only require a fraction of the river channel width and depth compared to traditional schemes, potentially allowing aquatic organisms to pass around, diminishing impact on biodiversity. Moreover, the open rotor design and relatively low rotational speed of VAT has been assumed to reduce the risk of fish collision^9,10^ both advantages when compared to Horizontal Axis Turbines (HATs).

Despite these apparent advantages of VAT, little is known about how cross-sectional confinement, flow condition, and associated hydrodynamic wake alterations might influence fish movement behaviour. In a confined marine field environment, benthic reef fish and larger predators avoided moving closer than 0.3 m and 1.7 m, respectively, away from a VAT rotor^9^. Similarly, brown trout were less likely to pass around a laboratory scale VAT, showing avoidance behaviour and awareness of the turbine^11^. Further behavioural observations reported include the lack of collisions between fish and VATs (blades and support structure) in field^9^ and laboratory^10,11^ tests. Behavioural adaptations in response to VAT, however, are rarely investigated in the context of flow alterations. Only Berry et al.^11^ reported that fish preferred the high-momentum regions on either side of a turbine compared to the low-momentum area of the turbine’s wake. Therefore, further research is required to understand the influence of environmental conditions, turbine presence and wake alterations on fish movement.

In this study, we explore how turbine operation state (i.e., stationary vs. rotating), discharge (i.e. mild vs. high), and cross-sectional confinement (i.e., confined vs. unconfined channel cross-section) influences the movement and behaviour of juvenile rainbow trout (*Oncorhynchus mykiss*; Walbaum, 1792), a wide-spread, partially invasive, freshwater salmonid species which can be found in most countries around the world^12^.

## Results

To investigate the effects of a standalone vertical axis turbine (VAT) under a range of flow conditions, operation states and flume widths on fish movement, we conducted experiments in two recirculating hydraulic channels of flume to turbine width of $w_{flume}/D$ = 2.5 (hereafter referred to as “narrow flume”) and 10 (hereafter referred to as “wide flume”) at the Cardiff University’s hydraulics facility. In both flumes, a similar test section was created, with a scaled three-bladed model VAT in its centre.

### Impact of discharge and turbine operation on fish movement

Using a simplified, semi-automatic motion tracking software and visual observations, the impact of flow condition [Mild (M) vs. High (H)] and turbine operation state [Stationary (S) vs. Rotating (R)] on fish swimming behaviour in a cross-sectional confined test section ($w_{flume}/D$ = 2.5, narrow flume) was analysed.

**Figure 1:**
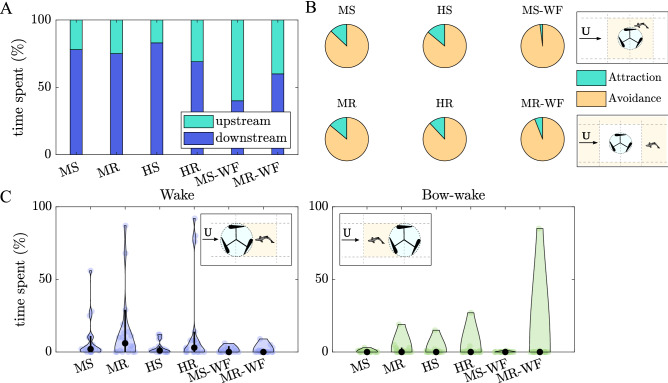
(**A**) Mean percentage of time spent upstream (green) and downstream (blue) of the turbine. (**B**) Percentage of time spent within the vicinity of the turbine ($-2\le x/D\le 2$, and $-1.25 \le y/D\le 1.25$ and $-2.5 \le y/D\le 2.5$ for the narrow ($w_{flume}/D=2.5$) and wide ($w_{flume}/D=10$) flume, respectively, with the area used for the analysis being scaled to account for the difference in flume width), termed as “attraction”, and outside this region, termed as “avoidance”. (**C**) Violin plots presenting the percentage of time spent within the wake ($0 \le x/D\le 5$, $-0.5 \le y/D\le 0.5$) and within the turbine’s bow-wake ($0 \le x/D\le -2$, $-0.5 \le y/D\le 0.5$) for each fish tested. Results are presented for each treatment, including MS, MR, HS and HR for the narrow flume and MS-WF and MR-WF for the wide flume.

**Figure 2:**
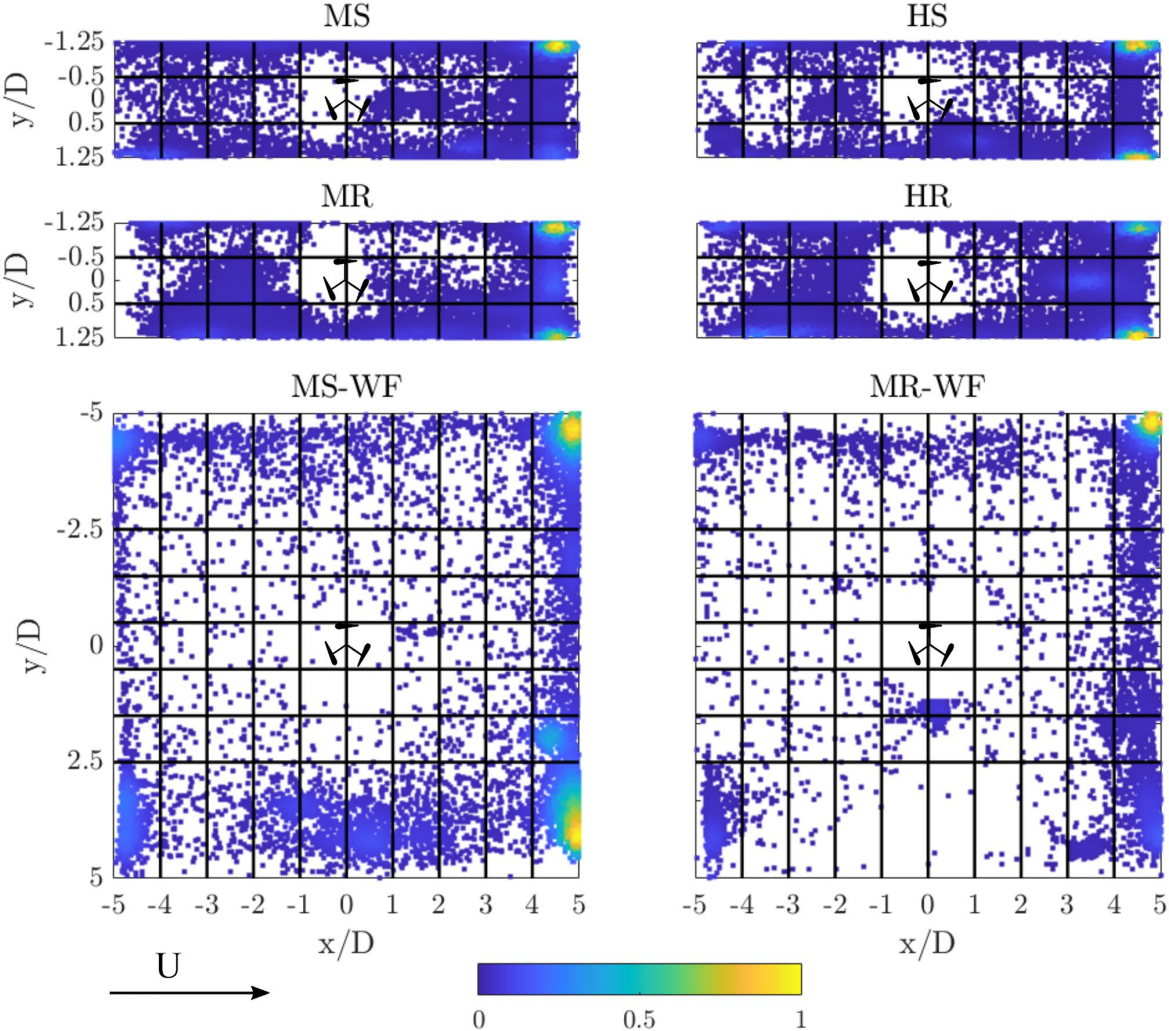
Scatter plot depicting the fish positions over the 10 min test time for all fish tested, contoured by the density of the scatter, with yellow marking the highest density of fish positions observed at a given location and blue colours showing lower densities. Results are presented for all four treatments analysed in the narrow flume (MS, MR, HS, HR) and both treatments investigated in wide flume (MS-WF, MR-WF). Note: MS-WF and MR-WF consists of fewer data points due to a reduced number of fish analysed and a lower frame rate used for the tracking analysis. Flow is from left to right.

Independent of the flow condition and turbine operation, fish spent most time downstream (MS: 78%, MR: 75%, HS: 83%, HR: 68%; GLM, *p* = 0.6395) and least upstream (MS: 22%, MR: 25%, HS: 17%, HR: 31%; GLM, *p* = 0.6395) of the turbine, as shown in Fig. 1A and indicated by an increase in scatter density in Fig. 2 which shows the fish position variation at a frequency of 1 Hz. Within the downstream section, fish preferred to stay near the side walls, particularly furthest downstream within the corners of the test section as highlighted by the yellow in the density scatter plot marking the fish positions over time (Fig. 2).

**Figure 3:**
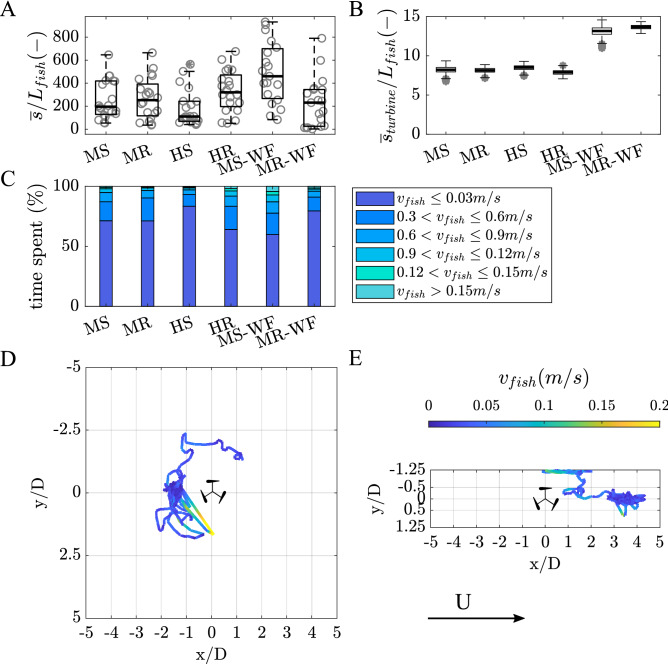
(**A**) Box plot showing the distribution of distances swam by each fish ($\overline{s}$), with the thick black line marking the median distance covered, the grey circles depicting the distance covered by each fish. (**B**) Box plot depicting the mean distance that fish maintained from the turbine ($\overline{s}_{turbine}$), with the thick black line marking the median and the the grey stars showing outliers. (**C**) Mean proportion of time spent at each velocity bin ($v_{fish}$), ranging from 0 to > 0.15 m/s. Results are presented for each treatment, including MS, MR, HS and HR for the narrow flume ($w_{flume}/D$ = 2.5), and MS–WF and MR–WF for the wide ($w_{flume}/D$ = 10). (**D**) and (**E**) Example movement trajectories showing a fish swimming in the turbine’s bow-wake (MR–WF) and the turbine’s wake (HR), respectively. Colour of the line indicated the swimming velocity $v_{fish}$ in m/s as shown in the colour bar. Flow is from left to right.

When the turbine rotated, fish spent slightly more time in the corner of the left-hand side of the test section (looking in the downstream direction; MS: 46%, MR: 28%, HS: 45%, HR: 25%) compared to the right-hand side (MS: 23%, MR: 33%, HS: 35%, HR: 29%). These observations, however, were not influenced by treatment (GLM, $p=0.2089$ and $p =0.7583$ for left and right-hand side, respectively). Given that most time downstream was spent along the flume walls, fish were infrequently observed swimming within the downstream centre (Fig. 1C; MS: 8%, MR: 14%, HS: 3%, HR: 15%) which corresponds to the low-momentum region of the turbine’s wake (see Fig. 6 in “Methods and materials” section). Although neither discharge nor turbine operation state significantly influenced the time spent within the wake (GLM, $p=0.195$), fish spent non-significantly more time in this region when the turbine rotated than when the turbine was stationary (Figs. 2 and 3E) which might be associated with lower stream-wise mean velocities and therefore a decrease in fish energy expenditure.

To analyse fish attraction and avoidance behaviour, the percentage of time spent in and outside the turbine’s vicinity is shown in Fig. 1B. Fish spent significantly more time avoiding the turbine (MS: 86%, MR: 86%, HS: 85%, HR: 88%; GLM, $p<0.001$) independent of turbine operation state and flow condition (GLM, attraction: $p=0.1950$, avoidance: $p=0.9658$). Although “attraction” time was influenced by the fish primarily using this region to move between the down- and upstream regions, fish were also observed to swim near the turbine or within the turbine’s bow-wake (Fig. 3D). During all treatments, however, only a few fish swam in the turbine’s bow-wake (Fig. 1C; mean percentage time: MS: 0%, MR: 2%, HS: 1%, HR: 2%, z-test; MS vs. MR: $p=1.000$, HS vs. HR: $p=1.000$, MS vs. HS: $p=0.4788$, MR vs. HR: $p=0.4996$), with the time spent within this region remaining unaffected by treatment (GLM, $p=0.6290$).

Correlating with the large proportion of time spent avoiding the turbine and time spent in the downstream corners of the test section, mean distance from the turbine ($\overline{s}_{turbine}$) was approximately 8–9 $L_{fish}$, as shown in Fig. 3B. Mean distance from the turbine was independent of flow discharge and turbine operation state (GLM, $p=0.8932$). Despite the larger proportion of time spent within the downstream corners, fish moved through the entire test section as depicted in Fig. 2. Mean distances covered ($\overline{s}$) were 257, 266, 185 and 331 $L_{fish}$ for MS, MR, HS and HR, respectively, as presented in Fig. 3A, with fish swimming significantly greater distances (1.8 times) under the HR compared to HS treatment (GLM, $p=0.0090$).

Movement behaviour was also investigated in terms of swimming velocity, with the distribution of swimming velocity and percentage of time spent swimming at different velocities presented in Fig. 3C. Independent of the treatment, fish spent most time holding station or slowly swimming (swimming velocities of 0–0.03 m/s; GLM, *p* = 0.08227). While fish swam for similar times at this velocity range under mild flow conditions when the turbine was stationary and rotating (MS and MR: 71%), under high flow conditions, they spent significantly more time at 0–0.03 m/s when the turbine was stationary compared to when the turbine rotated (HS: 84%, HR: 63%; GLM, *p* = 0.0114). In contrast, time spent swimming at velocities $\ge$ 0.15 m/s was significantly influenced by the discharge and turbine operation state (GLM, *p* = 0.0155). Specifically, a notable difference in swimming velocity was found when comparing HS and HR (GLM, *p* = 0.0068), and HR and MR (GLM, *p* = 0.0045). In contrast, turbine operation state did not impact on time spent swimming at velocities $\ge$ 0.15 m/s under mild flow condition (MR vs. MS; GLM *p* = 0.4928), and discharge was not an influencing parameter when the turbine was stationary (MS vs. HS; GLM, *p* = 0.5910).

**Figure 4:**
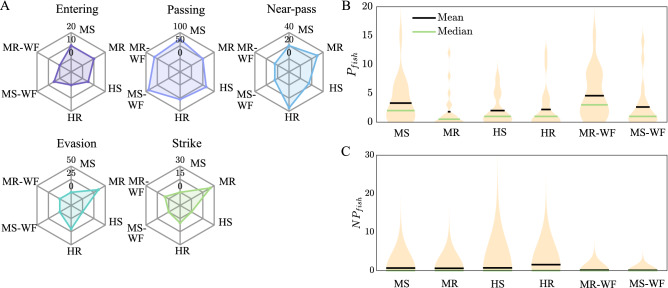
(**A**) Spyder plots depicting the percentage of fish observed to conduct the following behaviours: *Entering* into the turbine swept area; *Passing* from the downstream into the upstream section; *Near-pass*, i.e. attempting to pass into the upstream section but drifting or actively swimming downstream prior to passing *x*/*D* = 0; *Evasion*, i.e. sudden movement to avoid a collision with the turbine’s blade; and *Strike*, i.e. fish potentially colliding with the turbine’s blade. (**B**) Distribution of upstream passes per fish ($P_{fish}$) and (**C**) near-passes per fish ($NP_{fish}$) presented as violin plot with the black and green lines marking the mean and median of the distribution. Results are presented for all four treatments analysed in the narrow flume (MS, MR, HS, HR) and both treatments investigated in the wide flume (MS-WF, MR-WF).

Other fish behaviours analysed included entering into the rotor area, passing into the upstream region, attempting to pass (denoted as near-passes), actively evading a collision with the turbine, and experiencing a strike from one of the turbine blades. Fish entered the rotor area under all treatments except for the HR condition (MS: 10%, MR: 10%, HS: 5%, HR: 0%; z-test; MS vs. MR, HS vs. HR, and MS vs. HS, *p* = 1.000, MR vs. HR, *p* = 0.4682). Similarly, treatment did not impact the percentage of fish passing from the downstream into the upstream region (MS: 75%, MR: 50%, HS: 65%, HR: 55%; z-test, MS vs. MR: *p* = 0.1914, HS vs. HR: *p* = 0.7469, MS vs. HS: *p* = 0.7301, MR vs. HR: *p* = 1.000). In addition, the number of passes per fish ($P_{fish}$) were recorded as depicted in Fig. 4B, showing similar $P_{fish}$ for all treatments (mean ± s.d.; MS: 3 ± 4; MR, HS and HR: 2 ± 3; GLM, *p* = 0.5365). While a large number of fish successfully passed into the upstream section, others attempted to pass but drifted or actively swam downstream after reaching the centre of the test section (*x*/*D* = 0; red line in Fig. 4). Percentage of fish attempting to pass (MS: 20%, MR: 30%, HS: 15%, HR: 35%; z-test, MS vs. MR: $p=0.715$, HS vs. HR: $p=0.2733$, MS vs. HS and MR vs. HR: $p=1.000$) and likewise, near-passes per fish ($NP_{fish}$; GLM, $p=0.6189$; Fig. 4C) did not differ amongst treatments. Evasion moves were only observed when the turbine rotated (MS: 0%, MR: 35%, HS: 0%, HR: 20%), which were significantly higher under mild rather than high flow conditions (z-test, MS vs. MR: *p* = 0.0125, HS vs. HR: *p* = 0.1138, MR vs. HR: *p* = 0.4788). Likewise, potential collisions were only observed when the turbine rotated (MS: 0%, MR: 25%, HS: 0%, HR: 5%), with the percentage of fish potentially experiencing a strike being almost significantly affected by the discharge when the turbine rotated (MS vs. MR: *p* = 0.0558, HS vs. HR: *p* = 1.000, MR vs. HR: *p* = 0.184). No injuries were apparent and all fish remained active and continued swimming after a close contact with a turbine blade. Note, fish were not monitored after the experiments for external and/or internal injuries.

### Impact of lateral blockage on fish movement

The impact of spatial constriction was investigated by comparing fish swimming in the narrow flume (flume width: 0.3 m; treatments: MS and MR) versus wide flume (flume width: 1.2 m; treatment: MS-WF and MR-WF) under mild flow conditions and for both turbine rotation states [stationary (S) and rotating (R)] (see “Methods and materials” section). Here, the lateral length of the test section of the wide flume was four times the width of the narrow flume hence providing more space for fish to pass around the turbine, but with equal depth-averaged velocity and water depth. To account for the difference in lateral blockage, results non-dimensionalised by flume width ($w_{flume}$) are added in brackets for the distance maintained from turbine, $\overline{s}_{turbine}$, and the distance swam, $\overline{s}$).

While fish spent most time downstream and least time upstream under confined flume conditions (narrow flume), the increase in lateral space resulted in a higher percentage of time spent upstream independent of the turbine operation state (MS: 22%, MR: 25%, MS-WF: 60%, MR-WF: 40%) as shown in Fig. 1A, highlighting a significant impact of the spatial conditions (GLM, percentage time upstream: *p* = 0.0044, percentage time downstream: *p* = 0.0045). Particularly when the turbine was stationary (MS and MS–WF), time spent upstream and downstream significantly differed between the two flumes (GLM, *p* = 0.0012 and *p* = 0.0012, respectively). Downstream of the turbine, fish spent most time along the flume walls independent of the flume width, as shown in Fig. 2 (LHS: MS: 46%, MR: 28%, MS-WF: 27%, MR-WF: 48%, GLM, *p* = 0.1672; RHS: MS: 23%, MR: 33%, MS-WF: 12%, MR-WF: 11%, GLM, *p* = 0.0591). Hence, fish spent less time within the downstream centre of the test section as depicted in Fig. 1C (MS: 8%, MR: 14%, MS–WF: 1%, MR–WF: 1%; GLM, *p* = 0.2934) and Fig. 2. Fish length impacted time spent within the wake of the turbine, with larger individuals spending more time within the low-momentum region (GLM, estimate: 1.1228, std error: 0.2671, $p<$ 0.001; Fig. 6 in “Methods and materials” section).

Similar to the narrow flume test case, fish were also observed to swim within the turbine’s bow-wake in the wide flume (MS: 35%, MR: 40%, MS-WF: 5%, MR-WF: 11%). Spatial blockage did not significantly impact the number of fish observed swimming in the bow-wake when the turbine was stationary (MS vs. MS-WF; z-test, *p* = 0.0572) or when the turbine rotated (MR vs. MR-WF; z-test, *p* = 0.0818). While the time spent in the bow-wake greatly varied amongst individuals (Fig. 1C), percentage time spent was unaffected by fish standard length (GLM, *p* = 0.6961) and treatment (GLM, *p* = 0.5156).

Independent of turbine operation state and cross-sectional space (GLM, $p< 0.001$), fish spent significantly more time outside (MS: 86%, MR: 86%, MS-WF: 98%, MR-WF: 6%; GLM, *p* = 0.0942) than inside the turbine’s vicinity (MS: 14%, MR: 14%, MS-WF: 2%, MR-WF: 6%; GLM, *p* = 0.0962), indicating avoidance behaviour (Fig. 1C). Only when the turbine was stationary, time spent near the turbine significantly differed between flumes (MS vs. MS-WF; GLM, *p* = 0.0424).

On average, a greater mean swimming distance was apparent for MS–WF, which was almost twice the distance of the other treatments (MS: 257 $L_{fish}$ (50 $w_{flume}$), MR: 266 $L_{fish}$ (50 $w_{flume}$), MS–WF: 489 $L_{fish}$ (80 $w_{flume}$), MR–WF: 230 $L_{fish}$ (30 $w_{flume}$)), highlighting the significant impact of the cross-sectional confinement and turbine operation state (GLM, $p<0.001$) (Fig. 3A). More specifically, under unconfined conditions, fish covered a greater distance when the turbine was stationary rather than rotating (MS–WF vs. MR–WF; GLM, $p<0.001$). However, fish standard length did not significantly influence distance swam (GLM, *p* = 0.75421). Due to the increase in lateral space in the case of the wide flume and in agreement with the observations that fish spent most time within the downstream corners (Fig. 2), mean distance maintained from the turbine increased to 13 and 14 $L_{fish}$ (2 $w_{flume}$) for MS–WF and MR–WF, respectively, compared to 8 $L_{fish}$ (2 $w_{flume}$) for MS and MR (Fig. 3C). Hence, more space resulted in fish swimming greater distance from the turbine (GLM, MR vs. MR–WF: $p<0.001$ and MS vs. MS–WF: $p<0.001$) independent of the turbine operation state (GLM, MR vs. MS: *p* = 0.6623 and MR–WF vs. MS-WF: *p* = 0.1499). This was also influenced by fish standard length (GLM, *p* = 0.0043).

Swimming velocities were analysed by comparing the density distribution of the swimming velocities recorded and the percentage of time spent at these velocities (Fig. 3C, respectively). Fish held station or swam the most at swimming velocities of 0–0.03 m/s (MS: 71%, MR: 71%, MS-WF: 49%, MR-WF: 51%), unaffected by treatment (GLM, *p* = 0.0992) and fish standard length (GLM, *p* = 0.2397). While under confined conditions, fish spent equal time swimming at these velocities (MS and MR: 71%), under unconfined conditions, they spent significantly less time at the lower velocities when the turbine was stationary (MR–WF vs. MS-WF; GLM, *p* = 0.0134). In contrast, treatment significantly influenced the time spent at swimming velocities greater than 0.15 m/s (GLM, $p<0.001$) while fish standard length was a non-significant parameter (GLM, *p* = 0.5291). A significantly larger proportion of time was spent at higher velocities under unconfined conditions compared to confined conditions when the turbine was stationary (MS vs. MS-WF; GLM, $p<0.001$) and when the turbine rotated (MS-WF vs. MR-WF; GLM, $p<0.001$).

Fish entered the rotor region under all treatments except for the MR–WF condition (MS: 10%, MR: 10%, MS-WF: 5%, MR-WF: 0%; z-test, MS vs. MS-WF: *p* = 0.9793 and MR and MR-WF: *p* = 0.4908) (Fig. 4A). Strikes, on the other hand, were only observed when the turbine rotated, independently of the lateral blockage (MS: 0%, MR: 25%, MS–WF: 0%, MR-WF: 5%; z-test, MS vs. MS–WF: *p* = NA and MR vs. MR–WF: *p* = 0.2064). Likewise, evasion moves were only observed when the turbine rotated, with a significantly lower number of fish evading under unconfined conditions (MR: 25%, MR–WF: 5%; z-test, MR vs. MR–WF: *p* = 0.0151).

Upstream passes and near-passes were the most frequent observed behaviours. Independent of the turbine operation state and cross-sectional confinement, similar numbers of fish passed into the upstream section (MS: 75%, MR: 50%, MS–WF: 89%, MR–WF: 63%; z-test, MS vs. MS-WF: *p* = 0.4473 and MR vs. MR-WF: *p* = 0.6134) and attempted to pass upstream (MS: 20%, MR: 30%, MS-WF: 5% and MR-WF: 5%; z-test, MS vs. MS-WF: *p* = 0.3698 and MR vs. MR-WF: *p* = 0.1108). In agreement with the number of fish passing and attempting to pass, the number of passes per fish (MS: 20%, MR: 30%, MS-WF and MR-WF: 5%; GLM, *p* = 0.1325; Fig. 4B) and number of near-passes per fish (mean ± s.d.; MS: 1 ± 2, MR: 1 ± 1, MS-WF: 0 ± 1, MR-WF: 0; GLM, *p* = 0.7544; Fig. 4C) were unaffected by turbine operation state and cross-sectional confinement. Fish standard length, however, affected the number of near-passes per fish, with larger individuals being more likely to undergo passage attempts into the upstream region (GLM, estimate: 0.04049, std error: 0.01708, *p* = 0.0202).

## Discussion

As an alternative to hard-engineering infrastructure, small-scale hydropower by means of in-stream river turbines has the potential to generate sufficient decentralised, clean energy for remote communities. The presence of vertical axis turbines (VATs) and their changes in river flow hydrodynamics, however, may influence the unique aquatic environment by altering the movement of aquatic organisms. Therefore, we explored the impact of a single VAT on fish movement through a series of experiments, studying the influence of flow discharge, turbine operation state, and cross-sectional confinement.

Most importantly, while fish were released at the downstream end of the test section, turbine presence, operation, flow condition and cross-sectional confinement did not prevent them from passing into the upstream region. Under all test conditions, however, fish, showed avoidance behaviour of the close area around the turbine and its wake, and therefore, limited interaction with the turbine. Increasing the cross-sectional area of the test section, resulted in even greater distances kept from the turbine and more time spent outside the turbine’s wake. Behaviours observed (i.e., entering, passing, near-passing, colliding, evading), however, were unaffected by the cross-sectional confinement.

### Impact of discharge and turbine operation

The impact of discharge and turbine operation state was examined under cross-sectional confined conditions (narrow flume), which may naturally arise when positioning turbines in narrow river cross-sections, between rocks^9^, ducts^13,14^ or arrays^15,16^ to increase turbine performance.

Independent of the discharge and turbine operation state, fish spent most time within the downstream section, particularly along the sidewall and furthest downstream corners. Hence, only a few fish swam in the turbine wake. It should be considered that the large proportion of time spent downstream, however, may be influenced by fish being released within the downstream centre of the test section, fish seeking cover from predators and/or fish seeking shelter in the low-velocity regions near the flume walls and flow straighteners. This observation was contrary to what was expected as fish were previously observed to remain in the wake of a marine turbine support structure positioned in an unconfined, marine environment^17,18^. The limited attraction to the turbine wake may be a result of our low upper test discharge, constrained by the pump’s facility. Fish exposed to an even higher discharge environment (i.e., higher incoming flow) are anticipated to use low-momentum areas such as the turbine wake to seek refuge and save on energy expenditure. Additional experiments investigating fish spatial behaviour under higher flow conditions will be required to verify this hypothesis. Moreover, the low attraction to the turbine wake may be a result of the three-dimensional turbulent structures shed by the turbine blades, creating pockets of high turbulent kinetic energy and Reynolds shear stresses^16^ (Fig. 6). Previous studies showed fish avoiding regions of high vorticity, Reynolds shear stress and turbulent kinetic energy^19,20^, and highlighted the ability of coherent vortices to destabilise fish^19,21^. Exploiting the observed avoidance behaviour of turbulent wake regions may support diverting fish away from these obstructions within rivers, further minimising fish-turbine contact and subsequent injuries. Similar approaches using vortices to guide fish away from turbine entrances are currently investigated^22^. Nevertheless, further knowledge will be required to quantify the interactions between fish and these high energetic vortices, for instance, using particle image velocimetry, laser Doppler anemometry or numerical modelling (e.g., large-eddy simulation or machine learning^23^). Moreover, fish may avoid the turbine wake due to noise or sight but are not expected to avoid the turbine due to pressure changes. Changes to the pressure field due to the turbine are generated predominately by vortices shed in the turbine wake and not generated by changes in hydrostatic pressure and are therefore very small.

Interestingly, although non-significant, a small number of fish (5–40%) swam immediately upstream of the turbine in the bow-wake. So far, this behaviour has only been reported for stationary bluff obstacles such D-shape cylinders^24–26^. The bow-wake of a D-shape cylinder is characterised by low streamwise velocities and high-pressure^24^, whilst a turbine is a porous body that reduces the pressure drop in the flow direction. Rainbow trout swimming in this region swam with reduced tail-beat frequency, body wave speed, and body curvature, indicating a decrease in energy expenditure^24^. Although the bow-wake is characterised by a low fish Strouhal number (based on fish length), indicating sub-optimal swimming efficiency, the energy expenditure observed in this region suggests that “bow-waking” is a unique swimming mechanism to hold station, similar to Kármán gaiting^24^.

Besides potential avoidance effects associated with the turbine’s wake, fish avoided the turbine structure, as shown by the large distance kept from the turbine and most time spent outside its vicinity. Similarly, a laboratory study investigating the impact of a small-scale horizontal axis turbine (turbine diameter *D* = 0.25 m, tip-speed ratio TSR = 5, rotational speed of 20 rpm) on *Oryzias latipes*, *Gnathopogon elongatus*, and *Rhodeus ocellatus ocellatus* reported that 71% of the fish avoided the turbine area or swam away from the turbine^27^.

Despite avoiding the turbine and its wake, fish swam the streamwise length of the test section, including both downstream and upstream regions. While exploring the test section of the narrow flume, more than 50% of the fish passed at least once from downstream into the upstream region, unaffected by the treatment. Upstream passage was the most common behaviour, as also observed by Yoshida et al.^28^. Passage behaviour is species-specific but has so far only been studied for migratory fish species. Atlantic salmon (*Salmo salar*), for example, passed a model VAT more frequently than brown trout (*Salmo trutta*)^11^, and American shad (*Alosa sapidissima*)^10^. Berry et al.^11^ also highlighted that fish preferred to pass around the turbine rather than through the rotor area, consistent with the results presented here. In contrast, Castro-Santos and Haro^10^ reported that 72% of the downstream migrating Atlantic salmon smolts passed through, above, or underneath the VAT rotor in a confined flume ($w_{flume}/D$ = 2) while only 28% passed around the turbine, potentially associated with smolts being actively entrained or even attracted to the turbine. Passage behaviour around the turbine has been associated with fish body shape, with compressiform (torpedo shape) fish passing through the gap furthest away from the turbine compared to fusiform (tall and thin shape, small body width) fish^9^.

Another behaviour observed in the current study was fish entering the rotor area, which occurred during all treatments, except when the turbine rotated under high flow conditions (i.e., HR). Similar observations were reported for a helical, marine VAT installed between corals, showing that fish only entered the rotor area under low flow velocities and turbine rotational speeds^9^. Furthermore, the investigation of a ducted river turbine showed that 35% less fish entered the rotor area when the turbine rotated compared to stationary conditions^29^.

In the current study, potential collisions between individual fish and the turbine, and evasion movements to avoid a direct collision with the turbine blade were only observed by 5–25% and 0–35% of all individuals when the turbine rotated, respectively. Evasion movement has been observed across a wide range of species, characterised by burst swimming away from the turbine and sudden moves near the turbine blade^9^. The low number of close contacts observed here is consistent with the study by Hammar et al.^9^ and Berry et al.^11^ who reported no collision or close contacts with a model VAT. While collision risk is assumed to be influenced by wake alterations distracting fish from the turbine, under natural conditions, other environmental factors will also strongly influence strike risk (e.g., noise, turbidity)^28^. Proximity to the turbine may also depend on the fish’s personality^9,11^, with bolder, more explorative fish being closer to the turbine or even entering the rotor area, and shyer individuals keeping greater distances. Fish-turbine interaction (e.g., entering, colliding, avoiding) has also been related to fish swimming capabilities^27^. A laboratory study examining the impact of a scaled HAT showed that *Gnathopogon elongates* were more active near the turbine as their maximum swimming speed exceeded the turbine’s tip speed. In contrast, fish with slower swimming speeds or those with a preference for swimming near the bed such as *Oryzias latipes* and *Rhodeus ocellatus ocellatus* avoided swimming near the rotor, resulting in a reduced turbine interaction^27^.

### Impact of cross-sectional confinement on fish movement

While most laboratory studies investigated the impact of hydrokinetic turbines under cross-sectional confined laboratory conditions^10,11,27,28^, only a few studies have examined the influence of turbine cross-sectional confinement on fish movement^9^.

Despite being released at the downstream end of the test section, fish spent more time upstream in the wide flume. In the downstream section, fish spent most time near the flume walls, characterised by free-stream velocities. As in the confined conditions, fish spent least time within the turbine wake. Hence, fish preferred the free-stream region rather than the low-momentum region.

Likewise, fish spent less time within the turbine’s vicinity under unconfined, compared to cross-sectional confined conditions, further consolidating the assumption that fish avoid the near-turbine region. The difference in time spent within the turbine vicinity between the two confinement conditions likely arises from fish passing through this region to move between downstream and upstream sections in the narrow flume. The increased avoidance of the turbine region was also reflected by a greater distance maintained from the turbine under unconfined spatial conditions because of the increase in available swimming space. The increase in swimming space, however, did not result in an increase in swimming distance when the turbine rotated (MR-WF) but only when the turbine was stationary (MS-WF), indicating that lateral spacing is not the only dominant factor influencing fish swimming behaviour.

Although fish spent even less time in near-turbine region in the wide flume, similar numbers of fish entering the rotor area, potentially experiencing strikes, and showing evasion movements were observed. Similarly, an increase in the lateral cross-section did not increase the number of fish passing and attempting to pass upstream.

Consequently, an increase in lateral cross-section resulted in fish spending less time near the turbine or its wake indicating greater avoidance which, however, was not reflected in their behaviour. Due to the inconclusiveness of the results in this section, further research will be required to better understand the impact of lateral confinement on fish movement to define the ideal space needed between turbines within an array, or turbine and confinement (e.g., duct, rocks).

### Study limitations

Generalisation, transferability, and comparability of the fish behaviour experiments and the corresponding results to full-scale hydrokinetic VAT deployed in riverine, estuarine, and marine environments are limited. Fish behaviour studies were conducted under simplified, laboratory conditions with clean, purified water, disregarding the natural turbidity, for example, of rivers and estuaries. Water turbidity may be an important factor influencing fish vision and can influence fish behaviour. In addition, experiments were conducted under strong lighting conditions. To provide a comprehensive assessment of the impact of VAT on fish movement, further light, and turbidity conditions need to be tested, including, for instance, dark conditions^10,11^. Studies investigated HAT reported an increase in avoidance behaviour, which was characterised by more fish avoiding the turbine, swimming away, and less frequently approaching the turbine, under dark conditions^27^. This observed behaviour was assumed to be related to the inability to visually detect the blades^27^.

Another study limitation arises through the requirement of scaling of the experimental setup to the flume size available. While flow conditions and geometry of the investigated obstruction can be scaled using the Froude and Reynolds, and geometric scaling laws, respectively, determining the right fish size or scaling fish remains challenging. Allometric scaling, for example, may be applied to scale the size or mass of a fish according to morphological, physiological, and ecological traits^30^. Moreover, geometric scaling was found to appropriately scale length to volume, and surface to volume, assuming mass $\sim$ volume^31^. Adapting the size of a fish to match the scale of the experimental setup may result in the use of different life stages which, in turn, may result in considerable variations in behaviour responses depending on, for example, past experiences, noise, predators, feeding and hydrodynamics^32^. When using certain life stages, biological timing must also be considered.

Further limitations were caused by the experimental setup. To capture the whole of the test section, a single camera was positioned at a height conflicting with other laboratory installations, resulting in skewed and distorted images. To improve the swimming path and swimming speed analysis, a stereo vision camera system and/or a combination of top- and side-view cameras may be deployed, allowing the extraction of the 3D fish position. 3D tracks in combination with high-fidelity simulations or other advanced measurement techniques (e.g., PIV) to quantify the 3D flow field upstream and downstream of the turbine may be advantageous to extract swimming speeds, swimming modes (e.g., burst/sustained swimming, station holding, drifting) as well as swimming directions (e.g., actively swimming upstream/downstream).

## Conclusion

To analyse the potential of using vertical axis in-stream turbines (VAT), we investigated the impact of a single VAT on fish movement (juvenile rainbow trout) through a series of laboratory, scaled experiments, studying the influence of discharge, turbine operation state and cross-sectional confinement.

Our results showed that deploying a VAT under cross-sectional confinement did not prevent fish from moving between downstream and upstream section, despite having to pass through a high velocity region generated on either side of the turbine close to the turbine itself. Fish-turbine interactions were limited, indicating fish avoidance of the turbine. Similarly, fish spent limited time within the turbine’s wake, showing no attraction to the turbulent low-momentum wake region. Neither turbine operation state nor an increase in discharge changed the observed swimming behaviour under confined test conditions. Providing greater cross-sectional space around the turbine, showed a further reduction in time spent near the turbine and within its wake which was accompanied by the larger distance maintained from the turbine. Despite the enhanced avoidance of the turbine, similar behaviour was observed in terms of fish entering, passing, attempting to pass, colliding and evading the turbine as under confined test conditions.

Taking into account the observed avoidance fish behaviour of the turbine itself and its wake, turbine positioning within the main river channel and the lateral spacing between multiple turbines and their confinements should be carefully considered to preserve habitat connectivity and migratory routes. Moreover, further parameters potentially causing avoidance behaviour should be explored, including, for instance, turbine noise and visibility. Although fish were not prevented from passing the turbine under cross-sectionally confined test conditions, providing greater space around the turbine may further decrease fish-turbine interaction, potentially reducing the impact of the VAT on fish movement and the risk of fish collisions. Further research, however, will be required to develop guidelines for the suitable deployment of turbines in rivers (e.g, lateral and longitudinal spacing) to not compromise fish movement while achieving the highest possible energy generation.

## Methods and materials

Fish behaviour experiments were conducted at the Hydro-Environmental Research Centre’s hydraulics laboratory at Cardiff University (UK). All experiments were performed in accordance with relevant guidelines and regulationsand were approved by Cardiff University Animal Ethics Committee and conducted under Home Office License PPL 303424 following ARRIVE guidelines^33^.

### Experimental setup

**Figure 5:**
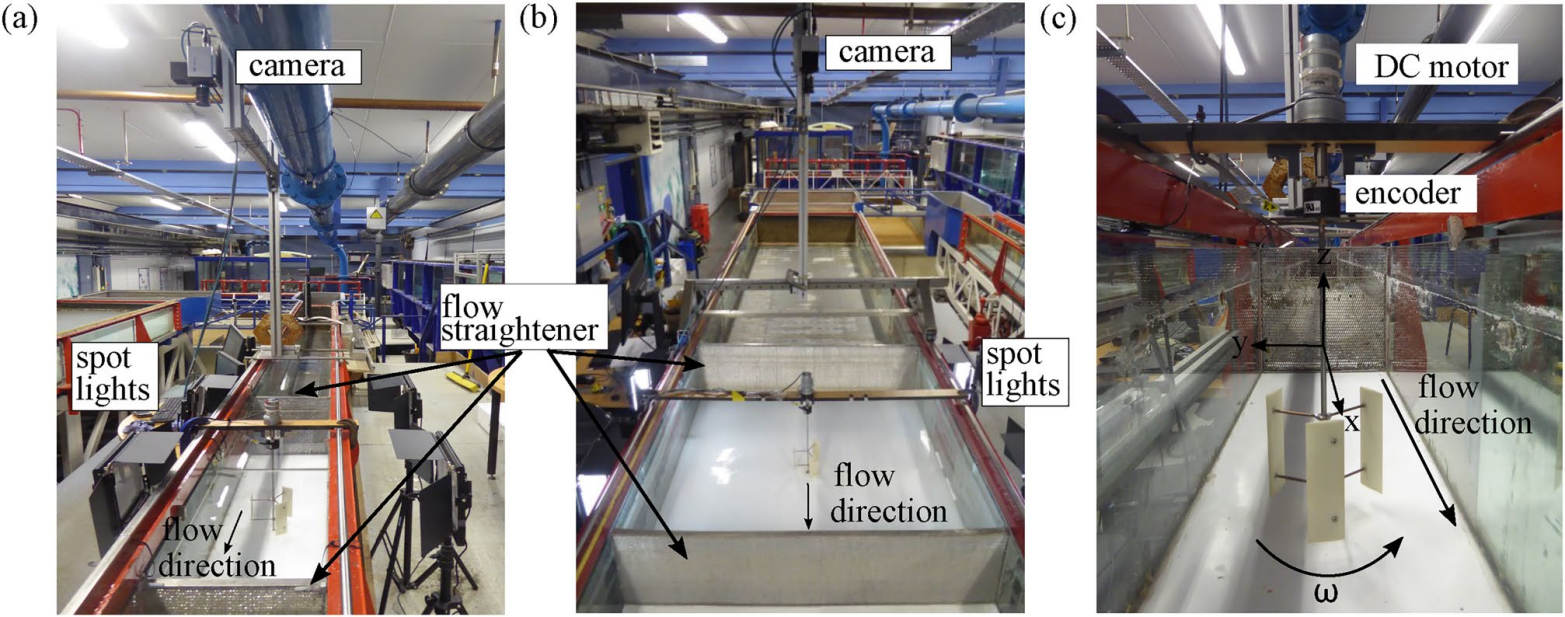
Photograph of the experimental setup in the narrow flume ($w_{flume}/D$ = 2.5) (**A**) and wide flume ($w_{flume}/D$ = 10) (**B**), depicting the streamwise location of a single vertical axis turbine of diameter *D*. The vertical axis turbine, shown in (**C**) was located within the lateral centre of the flume approximately 4.4 m downstream of the flume inlet, rotating in counter-clockwise direction; all photographs looking in upstream direction.

The first experiments were undertaken in a 10 m long, 0.3 m wide and deep recirculating flume with a longitudinal bed slope of 1/1000 (hereafter denoted as the narrow flume and referred to as “cross-sectional confined condition”; Fig. 5A). A three-bladed single VAT of height and diameter *D* = 0.12 m (Fig. 5C; further details can be found in Müller et al.^16^) was placed approximately 4.4 m downstream of the flume inlet and operated at optimum tip speed ratio of 1.9^34^. To connect the shaft with the flume bed and enhance the contrast between fish and flume bed, a white PVC board of approximately 10 mm thickness was attached to the flume bed, allowing the integration of a bearing to connect the turbine.

To examine the impact of cross-sectional confinement on fish, a second experiment was conducted in a flume of 10 m length, 1.2 m width and 0.3 m depth, with same bed slope (hereafter denoted as the wide flume [WF] and referred to as “cross-sectional unconfined conditions”; Fig. 5B). As in the narrow flume, a VAT of similar dimensions and rotational direction was placed approximately 4.4 m downstream of the flume inlet within the lateral centre of the flume. The bulk velocity and flow depth were kept constant in the narrow and wide flume to allow comparability between tests.

Both test sections were illuminated by two spotlights (Neewer Bi-Colour LED) positioned on either side of the test section, to minimise shaded areas and ensure equal light distributions. All experiments were conducted under ambient light conditions from LED lights mounted on the room ceiling.

To monitor fish swimming behaviour, a Baumer camera was mounted above the flume, recording monochrome series of tif images of size 600 $\times$ 2352 pixel and 2048 $\times$ 2000 pixel at approximately 80 fps and 55 fps for the narrow and wide flume, respectively. A 10 mm thick clear, transparent perspex plate was mounted on top of the water surface to prevent light reflections from interfering with the image quality. Additionally, a GoPro Hero camera (version 5, 7 and 9; 60 fps, 1080 $\times$ 1920 pixel), positioned on the left-hand side of the flume and mounted on a tripod, was used to simultaneously record the side view of the test section.

Experiments in the narrow flume ($w_{flume}/D$ = 10) were conducted for two flow conditions: denoted as “mild” (**M**; *Q* = 13 $ls^{-1}$; *h* = 0.23 m) and “high” (**H**; *Q* = 17 $ls^{-1}$; *h* = 0.23 m), and two turbine operational states: denoted as “stationary” (**S**; 0 rpm) and “rotating” (**R**; 58 and 75 rpm for 13 and 17 $ls^{-1}$, respectively). In contrast, experiments in the wide flume were only conducted under “mild” conditions. To ensure comparability between the two flumes, a flow depth of *h* = 0.23 m was kept in the wide flume whilst the discharge was adapted to *Q* = 53 $ls^{-1}$ to ensure a bulk velocity of *U* = 0.19 $ms^{-1}$ in both flumes. Discharge (*Q*) and flow depth (*h*) were regulated by a pump with 30 $ls^{-1}$ and 60 $ls^{-1}$ capacity for the narrow and wide flume, respectively, and a tailgate weir at the downstream end of each flume, respectively, and kept constant throughout the experiments. Flow depth was measured using a Vernier pointer gauge with an accuracy of  ± 0.1 mm, and discharge was measured with an ultrasonic flow meter (TecFluid Nixon CU100) with  ± 1.5% precision.

### Fish maintenance and holding facilities

Before and after the experiments, approximately 50 fish were held in 60–80 *l* tanks within a Recirculating Aquaculture System (RAS) at the Cardiff University Aquarium, enclosed within a temperature-controlled room maintained at 14 ± 0.5 °C on a 12h:12h dark–light cycle. This system has integrated bag and drum filters (Pall Cooperation) as well as a plastic bio media in the sump tank and an UV sterilization system. Water temperature and oxygen level were constantly monitored while nitrite levels were tested weekly, using a water quality test kit (Nutrafin). Fish were fed commercial trout pellets every morning.

Prior to the start of the experiments, fish were transported to a temporary holding tank at the hydraulic facilities at Cardiff University. The holding tank consisted of 500 *l* dechlorinated water (Seachem Prime Concentrated Conditioner, Tetra AquaSafe), constantly recirculated and chilled to 13 ± 1 °C (D–D The Aquarium Solution, DC 750) with an external filter (Aquamanta, EXF 600). The tank was aerated by multiple external air pumps (e.g., Tetratec Aps 400). Due to the manual operation of the ambient light, fish were maintained on a 14 h:10 h dark–light cycle. Depending on the experiment, fish were either maintained free swimming within the tank or in floating mesh cages.

All tanks and cages within the Cardiff University Aquarium and the hydraulic facilities were equipped with environmental enrichments to provide refugia (e.g., plant pots) and minimise stress. Particular care was taken to minimise stress when handling and transporting fish (max. 20 min journey) between facilities and tanks, with fish given at least 24 h recovery following transfer.

### Experimental procedure of fish experiments

On the test day, fish were introduced into the flume at the centre of the furthest downstream end of the test section and given a 20 min acclimatisation period. During this time, fish were allowed to explore the whole of the test section, while experiencing a 5 min incremental increase in discharge over the first 10 min up to the test discharge level (Mild = 13 $ls^{-1}$, High = 17 $ls^{-1}$), followed by a 10 min acclimatization at the test discharge. At all times, the downstream tailgate weir remained fixed at a pre-determined height, set for the uniform flow condition. After the acclimatisation, fish were caught using a fishing net and the perspex plate was mounted on top of the water surface. Then, fish were re-released at the most downstream end of the test section at the centreline of the main channel. Each trial lasted 10 min and 30 s. The additional 30 s added to the recording accounted for the handling and release of the fish as well as the cleaning of the perspex plate at the beginning of each trial and was excluded from the analysis, resulting in a total analysis time of *T* = 600 s.

During tests, human intervention was avoided when possible and only took place when fish impinged the fence, which often happened immediately after the release and within the first 30 s used to setup the trial. In this case, fish were carefully encouraged to swim or removed from the flow straightener by tapping against it. In the case of repeated impinging of the flow straightener, the experiment was terminated, and fish were removed from the test section and the analysis (indicted by number of excluded fish $N_{excluded}$).

After completion of the test (10 min 30 s), fish were weighed and measured, and then returned to the holding tank. At the end of each test series fish were transported back to RAS at the Cardiff University Aquarium.

### Study parameter

During the test, the following parameters were recorded and analysed:

#### Spatial usage

To analyse the spatial usage of the test section, each image series was converted into a video which was then analysed using JWatcher v.1.0. The test section was divided into 30 and 70 quadrants for the narrow and wide flumes, respectively. These quadrants were equally distributed between the upstream and downstream region. For each quadrant, the time spent ($t_{spent}$) was manually logged and the percentage of the total analysed time ($T=600$ s) was calculated.

The following parameters were determined based on the percentages of time recorded, including:

- *percentage of time spent upstream* (-5$\le x/D\le$0, narrow flume: $-1.25\le y/D\le 1.25$ or wide flume: $-5 \le y/D\le 5$) and *downstream* ($0 \le x/D\le 5$, narrow flume: $-1.25 \le y/D\le 1.25$ or wide flume: $-5 \le y/D\le 5$) of the turbine
- *percentage of time swimming in in the centre* ($0 \le x/D\le 5$, $-0.5 \le y/D\le 0.5$), *left* ($0 \le x/D\le 5$, narrow flume: $-1.25 \le y/D\le -0.5$ or wide flume: $-5 \le y/D\le -0.5$) and *right-hand* ($0\le x/D \le 5$, narrow flume: $0.5 \le y/D\le 1.25$ or wide flume: $0.5 \le y/D\le 5$) side of the downstream section
- *percentage of time swimming immediately upstream of the turbine* (here termed bow-waking; $-2 \le x/D\le -0.5$, $-0.5 \le y/D\le 0.5$)
- *percentage of time swimming in* ($-2 \le x/D\le 2$, narrow flume: $-1.25 \le y/D\le 1.25$ or wide flume: $-2.5 \le y/D\le 2.5$) and *outside* ($-2 \le x/D\ge 2$, narrow flume: $-1.25 \le y/D\ge 1.25$ or wide flume: $-2.5 \le y/D\ge 2.5$) of the turbine’s vicinity (here termed avoidance and attraction, respectively)

It should be noted that the area over which the time spent near the turbine (attraction) and away from the turbine (avoidance) was calculated and scaled for the wide flume to account for the increase in cross-sectional-space.

#### Swimming distance

Fish position was extracted using Kinovea v0.8.15, a semi-automatic, open-source tracking software^35^. Due to the high susceptibility to errors (e.g., shaded areas, fish swimming close to flume walls, particles drifting through the test section), videos were created using a reduced frame rate of 2 fps and 1 fps for the narrow and wide flume, respectively, to reduce such errors through manual correction of the frames. For each frame, streamwise (*x*) and lateral (*y*) position of the fish was recorded and extracted from the software. Then, data were analysed using MS Excel and Matlab 2019a,b and 2020a. Prior to the analysis, the extracted fish positions were scaled to account for calibration errors. Based on the corrected coordinates, the total distance covered ($\overline{s}$) by each fish was estimated using the Pythagorean theorem. Similarly, the mean distance between fish and turbine ($\overline{s}_{turbine}$) was estimated over time.

#### Swimming velocities

Swimming velocities were determined based on the distance covered (as described in (ii)) between frames and the corresponding time step ($v_{fish}=s/\Delta t$). This approach provides an estimate of the swimming velocities over time but neglects the direction of the fish (i.e., whether the fish actively swam or drifted downstream), swimming depth and local flow velocity as wake velocities were only recorded for the MR-WF treatment. Based on the calculated velocities, the time spent at predefined swimming velocity ranges was calculated.

#### Fish behaviour

A range of behaviours were defined and recorded while analysing the data sets^28,36^. The investigated behaviours are described and visualised in Table 1. Additionally, the short video clips provided depict the individual behaviours. For simplification purposes, number of upstream passes per fish and percentage of upstream passes per fish were summarised under the term “passage behaviour”. Similarly, the term “movement behaviour” was also used, which refers to the distance covered and maintained from the turbine as well as the range of swimming velocities observed.

**Table 1:** Summary of behaviours recorded, including passing into the upstream section, attempting to pass, entering the rotor area, evading to prevent a contact with the turbine blade, and strikes^28,36^.

Behaviour	Description	
Passing	Movement from the downstream section into the upstream section using *x*/*D* = 0 as cut-off point	
Near-pass	Swimming towards the cut-off line at *x*/*D* = 0 followed by drifting or actively swimming downstream	
Entering	Fish entering into turbine swept area (light blue circle)	
Evasion	Sudden change in swimming direction in close proximity to the turbine to avoid a direct contact with the turbine	
Strike	Fish potentially experiencing contact with the turbine’s blade. Each behaviour was derived from the count of an individual event and the percentage of fish showing this behaviour	

### Experimental studies

In total, two experimental studies were conducted, investigating the impact of a single VAT on individual juvenile rainbow trout *Oncorhynchus mykiss*, Walbaum 1792), sourced from the Bibury Trout Farm, UK, and chosen as generic model species.

#### Narrow flume study

In a first study conducted in the narrow flume, the effect of discharge and turbine operation state on fish movement was investigated by exposing individual fish to two discharges (mild: *Q* = 13$ls^{-1}$ and high 17 $ls^{-1}$) and two turbine operation states (rotating and stationary) under confined test condition in the narrow flume. The range of test combinations resulted in the following four treatments: MS (mild-stationary), MR (mild-rotating), HS (high-stationary), and HR (high-rotating).

Fish passage behaviour tests were conducted between 23 November and 1 December 2020 between 8 am and 5 pm. For each treatment, $N_{tested}$ = 20 juvenile rainbow trout were tested, resulting in a total of 80 fish of mean standard length ± s.d., 57.0 ± 5.9 mm, mean total length ± s.d., 66.8 ± 6.9 mm, and mean mass ± s.d., 3.1 ± 0.9 g. An overview of the number of fish tested ($N_{tested}$), excluded ($N_{excluded}$) and analysed ($N_{analysed}$) as well as mass (*m*), standard ($L_{fish}$) and total ($L_{fish, total}$) length is provided in “Methods and materials” section. Fish tested for each treatment did not significantly differ in fish standard length (GLM, *p* = 0.7904), total length (GLM, *p* = 0.5691) and mass (GLM, *p* = 0.1281).

Treatment order was not randomised as each fish was only exposed to a single treatment. The MS treatment was tested first, followed by MR, HS, and HR. For each test, the parameters listed in “Methods and materials” section were analysed using the image series obtained.

Statistical analysis was conducted using R v.3.6.3 statistical software. Using a Gaussian General Linear Models (GLM), the time upstream and downstream was tested for significance with an identity and inverse link function, respectively. A Gaussian GLM with identity link function was also used to examine the proportions of time spent bow-waking, within the downstream centre as well as in the left and right-hand side of the downstream section; attraction and avoidance time; the time proportion within and outside the turbine’s vicinity; the proportions of time spent swimming at a range of different swimming speed; and the difference in distance swam and distance maintained from the turbine and the treatments. Likewise, the differences in fish standard length, total length and mass amongst treatments was tested for significance using Gaussian GLMs with identity link. As these parameters did not significantly differ amongst treatments, fish standard length was not included as an independent parameter in the final models. Hence, treatment was the only independent variable. Moreover, a two-proportion z-test was applied to examine the significant difference between treatments for the percentage of fish passing upstream, performing near-passes, bow-waking, entering, showing evasion behaviour, or experiencing strikes. Non-significant variables were stepwise removed from the statistical analysis and residuals were used to assess the suitability of the tests. *p* value significance was taken as 0.05.

#### Wide flume experiment

Here, the impact of cross-sectional confinement was tested by conducting a replica test in the wide flume. The test section in this flume had four times the width compared to the test section in the narrow flume. The impact of flow blockage was analysed for both turbine operation states (stationary and rotating) and the mild flow condition. These treatments are termed MS-WF and MR-WF.

Fish behaviour experiments for this study were conducted between 29 March and 7 April 2021 between 8am and 5pm. In total, 22 and 21 fish were tested for MS–WF and MR–WF, respectively. Due to technical difficulties (e.g., incomplete recordings) and fish impinging the downstream flow straightener, $N_{excluded}$ = 5 fish were excluded from the analyses, resulting in 19 fish per treatment and therefore, a total number of $N_{analysed}$ = 38 fish of mean standard length  ±  s.d., 50.0 ± 3.4 mm, mean total length ± standard deviation, 57.7 ± 3.2 mm, and mean mass, ± s.d., 2.0 ±  0.4 g. An overview of the number of fish tested ($N_{tested}$), excluded ($N_{excluded}$) and analysed ($N_{analysed}$) as well as mass (*m*), standard ($L_{fish}$) and total ($L_{fish, total}$) length is provided for each treatment in “Methods and materials” section. Fish tested for each treatment did not significantly differ in fish standard length (GLM, *p* = 0.1658), total length (GLM, *p* = 0.1926) nor mass (GLM, *p* = 0.0642).

As in the narrow flume experiment (“Methods and materials” section), treatment order was not randomised as each fish was only exposed to a single treatment. The MS-WF treatment was tested first, followed by MR-WF. For each test, the parameters listed in “Methods and materials” section were analysed using the image series obtained.

As the focus of this experiment was a comparison of cross-sectional confined and unconfined conditions for fish movement, results from MS-WF and MR-WF were compared against the results obtained in the narrow flume experiment for MS and MR. Due to the significant difference in fish standard length (GLM, $p<0.001$), total length (GLM, $p<0.001$) and mass (GLM, $p<0.001$) between the fish used in the narrow flume and wide flume experiment, fish standard length was included as an independent variable alongside treatment. The standard length of the fish used in the wide flume experiment was significantly smaller than the ones tested in the narrow flume experiment (GLM, MR vs. MR-WF: $p<0.001$, MS vs. MS-WF: $p<0.001$). The difference in standard length, total length, and mass for MS-WF and MR-WF, and for MS, MR, MS-WF and MR-WF were determined using a Gaussian GLM with identity link function. As in the narrow flume experiment, the significant impact of treatment and standard length on parameters based on the proportion of time spent (i.e., upstream, downstream, downstream centre, left and right-hand side, in and outside the turbine’s vicinity, swimming velocities) was examined using Gaussian GLMs with identity link function. Similarly, attraction and avoidance time were compared using a Gaussian GLM with identity link function. Distance covered during the test period and mean distance kept from the turbine were tested for significance using Gaussian GLMs with identity and inverse link function, respectively. A two-proportion z-test was used to compare the impact of spatial blockage depending on turbine operation state on the number of upstream passing fish, fish attempting to pass, bow-waking, entering the turbine, evading, and experiencing a strike. Non-significant variables were stepwise removed from the statistical analysis and residuals were used to assess the suitability of the tests. P-value significance was taken as 0.05.

### Fish measurements

Table 2 provides and overview of the number of fish tested ($N_{tested}$), excluded ($N_{excluded}$) and analysed ($N_{analysed}$) as well as mass (*m*), standard ($L_{fish}$) and total ($L_{fish, total}$) length for each treatment tested in the narrow flume (MS, MR, HS, HR) and 2 (MS-WF, MR-WF).

**Table 2:** Fish behaviour experimental details comprising number of tested fish ($N_{tested}$), excluded ($N_{excluded}$) and analysed ($N_{analysed}$) as well as their average mass (*m*), standard ($L_{fish}$) and total length ($L_{fish, total}$)  ±  s.d. for each treatment and experimental setup.

Treatment	$$N_{\begin{array}{c} tested \end{array}}$$	$$N_{\begin{array}{c} excluded \end{array}}$$	$$N_{\begin{array}{c} analysed \end{array}}$$	*m* $\pm s.d.$ (g)	$L_{fish}$ $\pm s.d.$ (mm)	$$L_{\begin{array}{c} fish, total \end{array}}$$ $\pm s.d.$ (mm)
MS	20	0	20	3.4 ± 0.9	57.0 ± 5.7	67.9 ± 7.8
MR	20	0	20	2.9 ± 0.7	56.2 ± 5.3	65.7 ± 5.8
HS	20	0	20	2.9 ± 0.8	56.6 ± 6.1	65.8 ± 6.7
HR	20	0	20	3.3 ± 0.9	58.0 ± 6.8	68.0 ± 7.4
MS-WF	21	2	19	1.9 ± 0.3	49.2 ± 2.1	57.0 ± 3.3
MR-WF	22	3	19	2.1 ± 0.4	50.7 ± 4.3	58.4 ± 3.1

### Fish videos

Videos visualising the observed swimming behaviours are provided, including a video clip showing entering, evading, near-passing, passing, close contact, swimming in the wake and the turbine’s bow wake. All video clips are top-view recordings with the flow from right to left. Only the video showing a fish entering the turbine swept area was recorded using a side-view camera, with flow from right to left.

### Hydrodynamic measurements

Complementary to the fish behaviour experiments presented, extensive hydrodynamic measurements were undertaken to quantify the three-dimensional wake effects of a single VAT. Using acoustic Doppler velocimetry, 10 downstream cross-section were measured within the y-z-plane for a similar experimental setup and hydraulic conditions as described for the wide flume (solely excluding the white background). A detailed description of the methodology is provided by Müller et al.^16^.

**Figure 6:**
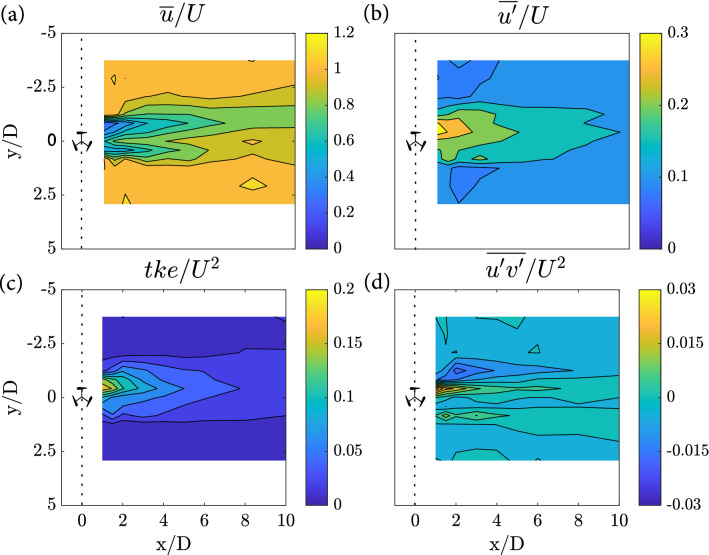
Horizontal planes at mid-turbine height (*z*/*D* = 0.67) showing contours of normalised (**A**) streamwise mean velocities, (**B**) streamwise turbulence intensity, (**C**) turbulent kinetic energy and (**D**) horizontal Reynolds shear stress, downstream of single rotating VAT under mild flow conditions (i.e., MR). Flow is from left to right.

Here, an exemplary cross-section is presented, depicting the *yz*-plane at mid-turbine height for (a) streamwise mean velocities normalised by *U* = 0.19 ${\textrm{m}}\,{\textrm{s}}^{-1}$, (b) streamwise turbulence intensity normalised by U, (c) turbulent kinetic energy normalised by $U^{2}$ and (d) horizontal Reynolds shear stress normalised by $U^{2}$ (Fig. 6).

Our results show a region of low-momentum velocity immediately downstream of the turbine rotor. This region was particularly pronounced at the upstroke side where the blades moved against the flow (i.e., left-hand side of the flume when looking in downstream direction), causing the wake to be asymmetric about its centerline^37–39^. Besides the reduction in mean streamwise velocity (a) at the upstroke side, the wake of a single VAT was characterised by high levels of $\overline{u}'/U$, $tke/U^{2}$, and $\overline{u'v'}/U^{2}$. The increase in turbulence in this region is a result of the shedding of energetic vortices^40,41^ which, however, only prevailed within the near wake ($x/D<2$)^34^. The downstroke side (i.e., right-hand side of the flume), in contrast, was characterised by lower turbulence levels. Following the near wake ($x/D<3$), the wake laterally expanded within the transition region (3$<x/D<5$) and almost fully recovered by $x/D\ge 6$ within the far wake, reaching velocity levels close to the approach flow velocity.

A more detailed analysis of the wake hydrodynamics of a single VAT can be found in Müller et al.^16^.

## Supplementary Information

Supplementary Legends.

Supplementary Video 1.

Supplementary Video 2.

Supplementary Video 3.

Supplementary Video 4.

Supplementary Video 5.

Supplementary Video 6.

Supplementary Video 7.

## Data Availability

The datasets generated and/or analysed during the current study are available in the Cardiff University data repository: http://doi.org/10.17035/d.2022.0220288116.
