# Supplementary material for: Fish response to the presence of hydrokinetic turbines as a sustainable energy solution
Source: Sci Rep. 2023 May 8;13:7459. doi: 10.1038/s41598-023-33000-w (PMC10167261; doi:10.1038/s41598-023-33000-w)
Supplement: Supplementary file 1 — Supplementary Legends. [file 41598_2023_33000_MOESM1_ESM.docx]

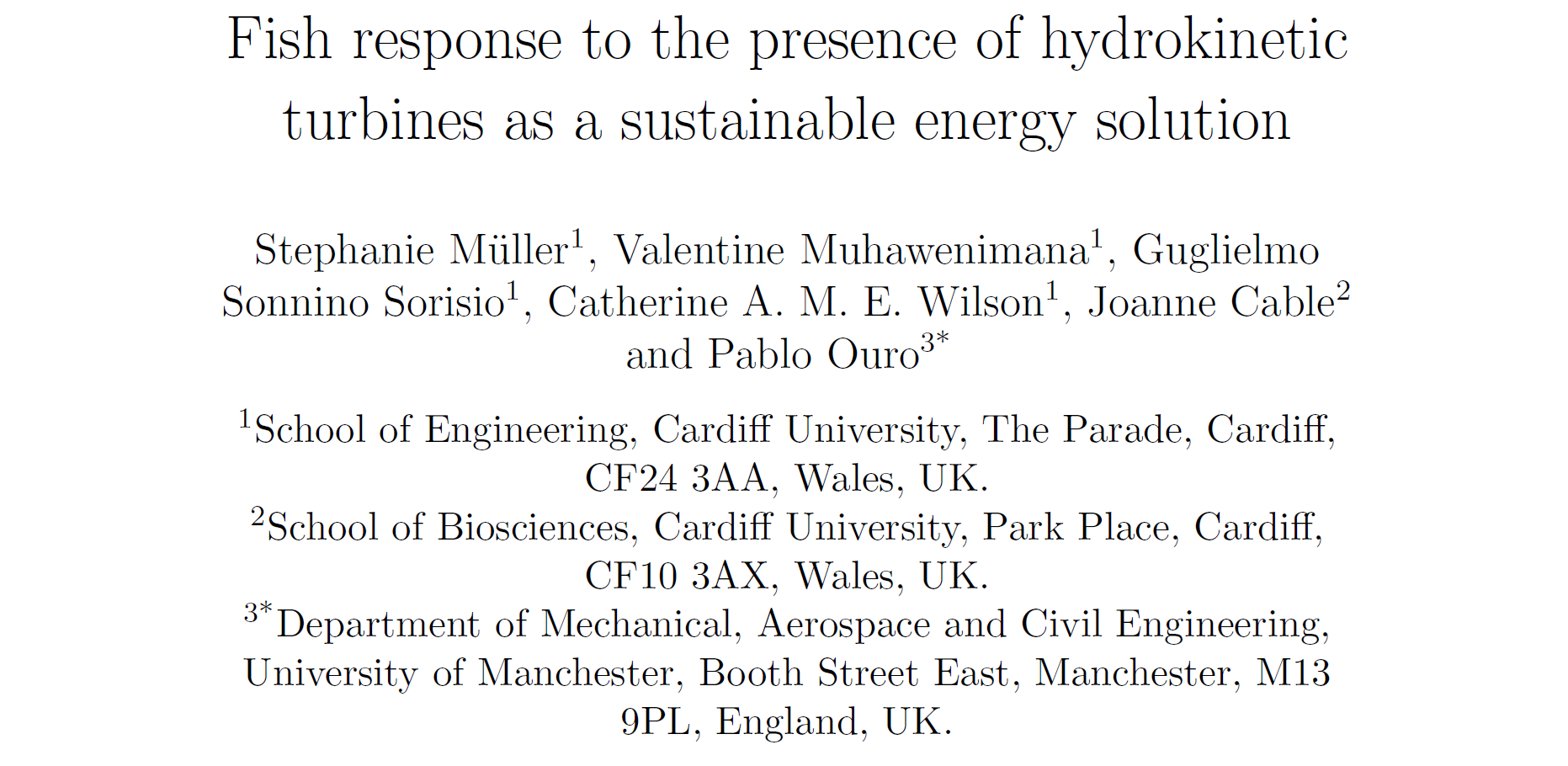


Video 1: Recording of a fish entering the swept area of a turbine that was stationary.

Video 2: Recording of a fish evading collision with the blades of the rotating turbine.

Video 3: Recording of a fish moving from down- to up-stream of the flume passing closely to a rotating turbine.

Video 4: Recording of a fish moving from down- to up-stream of the flume passing over the side of a rotating turbine.

Video 5: Recording of a fish that moving towards a rotating turbine closely enough to almost collide with it.

Video 6: Recording of a fish swimming in the wake of a rotating turbine.

Video 7: Recording of a fish swimming in the bow wake of a rotating turbine.
